# Interactive Effects of Pesticides and Nutrients on Microbial Communities Responsible of Litter Decomposition in Streams

**DOI:** 10.3389/fmicb.2018.02437

**Published:** 2018-10-17

**Authors:** Florent Rossi, Stéphane Pesce, Clarisse Mallet, Christelle Margoum, Arnaud Chaumot, Matthieu Masson, Joan Artigas

**Affiliations:** ^1^Laboratoire Microorganismes: Génome et Environnement, CNRS, Université Clermont Auvergne, Clermont-Ferrand, France; ^2^Irstea, UR RiverLy, Centre de Lyon-Villeurbanne, Villeurbanne, France

**Keywords:** microcosm, stressors interaction, leaf litter, fungal communities, microbial ecotoxicology, macroinvertebrates

## Abstract

Global contamination of streams by a large variety of compounds, such as nutrients and pesticides, may exert a high pressure on aquatic organisms, including microbial communities and their activity of organic matter decomposition. In this study, we assessed the potential interaction between nutrients and a fungicide and herbicide [tebuconazole (TBZ) and S-metolachlor (S-Met), respectively] at realistic environmental concentrations on the structure (biomass, diversity) and decomposition activity of fungal and bacterial communities (leaf decay rates, extracellular enzymatic activities) associated with *Alnus glutinosa* (*Alnus*) leaves. A 40-day microcosm experiment was used to combine two nutrient conditions (mesotrophic and eutrophic) with four pesticide treatments at a nominal concentrations of 15 μg L^-1^ (control, TBZ and S-Met, alone or mixed) following a 2 × 4 full factorial design. We also investigated resulting indirect effects on *Gammarus fossarum* feeding rates using leaves previously exposed to each of the treatments described above. Results showed interactive effects between nutrients and pesticides, only when nutrient (i.e., nitrogen and phosphorus) concentrations were the highest (eutrophic condition). Specifically, slight decreases in *Alnus* leaf decomposition rates were observed in channels exposed to TBZ (0.01119 days^-1^) and S-Met (0.01139 days^-1^) than in control ones (0.01334 days^-1^) that can partially be explained by changes in the structure of leaf-associated microbial communities. However, exposition to both TBZ and S-Met in mixture (MIX) led to comparable decay rates to those exposed to the pesticides alone (0.01048 days^-1^), suggesting no interaction between these two compounds on microbial decomposition. Moreover, stimulation in ligninolytic activities (laccase and phenol oxidase) was observed in presence of the fungicide, possibly highlighting detoxification mechanisms employed by microbes. Such stimulation was not observed for laccase activity exposed to the MIX, suggesting antagonistic interaction of these two compounds on the ability of microbial communities to cope with stress by xenobiotics. Besides, no effects of the treatments were observed on leaf palatability for macroinvertebrates. Overall, the present study highlights that complex interactions between nutrients and xenobiotics in streams and resulting from global change can negatively affect microbial communities associated with leaf litter, although effects on higher trophic-level organisms remains unclear.

## Introduction

Freshwaters pollution resulting from human activities has become a widespread phenomenon over the last decades. Among pollution sources, agriculture intensification coupled with changes in practices to increase agricultural yields have led to the massive inputs of both nutrients (including nitrogen and phosphorus) and toxicants (including pesticides which are generally used in mixture) in many stream ecosystems (Trewavas, 2002; Vörösmarty et al., 2010). Accordingly and despite the effort of the European Water Framework directive (WFD) to improve surface water chemical quality, more than 56% of streams in Europe are still qualified as moderate ecological status or worst regarding chemical contamination (WISE WFD database, 2017). This reality copes with the still increasing fertilizers (including NO_3_, P_2_O_5_ ad K_2_O) and pesticides consumption across Europe (FAOSTAT). In this context, the evaluation of the threat posed by such a chemical multi-contamination on aquatic organisms and stream ecosystems integrity and the ecological functions and services they provide has become a central preoccupation for societies, stream managers and scientists (Brosed et al., 2016).

Among aquatic organisms that can be found in headwater stream ecosystems, heterotrophic microbial communities have a pivotal role. Mainly composed by fungi (representing up to 98% of the total microbial biomass), microbial communities in leaves also host viruses, bacteria and protists. Thanks to a large panel of extracellular enzymatic activities (Chandrashekar and Kaveriappa, 1991; Abdel-Raheem and Ali, 2004), including ligninolytic (laccase, phenol oxidase) and celluloytic (β-glucosidase, cellobiohydrolase) enzymes, fungi and bacteria directly participates to the mineralisation of leaf litter (up to 45% of the total leaf mass loss). Thus, they have an important role in the recycling of organic matter in stream ecosystems (Cummins, 1974; Gessner and Chauvet, 1994; Webster and Meyer, 1997). In addition, microorganisms have been shown to stimulate leaf consumption by macroinvertebrates by (i) softening leaf tissues through their extracellular enzymes activity and ii) increasing the nutritional quality and palatability of the leaves (Suberkropp et al., 1983; Baldy et al., 2007). Overall, heterotrophic microbial communities play a non-negligible role in many biogeochemical cycles in addition of being at the base of the trophic networks in stream ecosystems (Webster and Meyer, 1997).

However, pesticides inputs may cause serious threat on such communities, possibly impairing microbial decomposition activity, thus, unbalancing the entire food web. This is particularly the case of fungicides, whose direct effects on fungi, including aquatic hyphomycetes, have already been described (Yang et al., 2011). In polluted streams, one of the most frequently detected fungicide is the tebuconazole (TBZ) (e.g., 11% of French rivers, NAIADES database, EauFrance, 2010–2015). Belonging to the azole family, TBZ (C_16_H_22_ClN_3_O) is a triazole fungicide (Richardson, 2009) that inhibits the sterol C-14 α-demethylation of 24-methylenedihydrolanosterol (which is a precursor of ergosterol, a key component of the fungal cell membrane) thus limiting the development of fungal biomass (Copping and Hewitt, 2007). Accordingly, it was previously shown that TBZ exposure can have negative effects on biomass, structure and extracellular enzymatic activities of fungal communities, overall leading to a decrease in the decomposition rates of leaf litter (Bundschuh et al., 2011; Zubrod et al., 2011, 2015; Artigas et al., 2012). Moreover, indirect effects of tebuconazole have been also shown to affect higher trophic level organisms such as macroinvertebrates due to the decrease of leaf nutritional quality (i.e., decrease in fungal biomass and/or changes in microbial community structure), suggesting a potential influence at the stream food web (Bundschuh et al., 2011; Zubrod et al., 2011). In addition to the fungicides, heterotrophic microbial communities are also chronically exposed to herbicides which are prevalent in these ecosystems. The most frequently detected herbicide in river ecosystems since the last 10 years is the S-metolachlor (S-Met) (e.g., 24% of French rivers, NAIADES database, EauFrance, 2010–2015). Belonging to the chloroacetanilide family, S-Met (C_15_H_22_ClNO_2_) is the S isomeric form of the herbicide metolachlor. Mostly used for maize treatment, this herbicide inhibits the fatty acid elongation 1 (FAE1) synthase, a key enzyme involved in the elongation of very-long fatty acids in plants (Götz and Böger, 2004; Paul et al., 2006). Although its effects on aquatic heterotrophic microbial communities colonizing leaf-litter are unknown, S-Met has been shown to induce changes in the structure of diatom communities accompanied with frustule deformation at concentrations ≥ 5 μg L^-1^ (Roubeix et al., 2011) and to inhibit cell reproduction of the green algae *Scenedesmus vacuolatus* by 50% after a 6h exposure to very high concentrations (598 μg L^-1^).

Given the fact that both pesticides and nutrients generally occur at the same time in stream ecosystems (Pesce et al., 2008), it appears essential to assess their interaction in order to understand the consequences at the microbial community level (Segner et al., 2014). Only a few studies tested the interactive effects between nutrients and fungicides on leaf-associated microbial communities, and the available knowledge suggest that the microbial stimulation by addition of nutrients can compensate fungicide toxicity (Fernández et al., 2016; Gardeström et al., 2016). Similarly, some studies tested the interactions between two different types of pesticides on leaf-associated microbial communities (Flores et al., 2014; Dawoud et al., 2017). In the case of the fungicide imazalil and the insecticide diazinon, Flores et al. (2014) observed that the effect of a mixture of both pesticides led to similar effects than the fungicide alone. On the other hand, (Dawoud et al., 2017) demonstrated that both insecticide (lindane) and fungicide (azoxystrobine) exposure lead to a stimulation of fungal sporulation, but that this stimulation was not observed in the presence of both pesticides in mixture. Together, these results highlights that interactions, such as synergism, between different pesticides may occur, although this seems tightly linked to the type of pesticide compound tested as well as their concentrations. Besides, such differences could also be explained by differences in terms of nutrient concentrations in the media, as (Flores et al., 2014) used stream water whereas Dawoud et al. (2017) used standard test medium M7 (OECD, 1998) for their exposure conditions. However, to our knowledge, no study evaluated the interactions between nutrients and a cocktail of different types of pesticides on leaf-associated microbial communities and the implication of such exposure on the palatability of leaves for macroinvertebrates.

Accordingly, the present study aims to assess (i) the interactions between nutrient increase, namely nitrate and phosphate, relative to stream eutrophication and the presence of TBZ and S-Met alone or in mixture on microbial decomposer communities associated to submerged leaves and (ii) how such multi-contamination further affect leaf palatability for macroinvertebrate consumers. In order to answer these two objectives, we conducted a microcosm experiment in a 2 × 4 full factorial design using laboratory artificial streams, with nutrient condition as the first factor (mesotrophic versus eutrophic) and pesticide treatment as the second one (control, TBZ alone; 15 μg L^-1^, S-Met alone; 15 μg L^-1^ and MIX; 15 μg L^-1^ of each compounds) over 40 days exposure. Chosen concentrations of pesticides were based on values observed on the field (Berenzen et al., 2005; Kalkhoff et al., 2012) and already used in laboratory experiments (Artigas et al., 2012; Donnadieu et al., 2016; Pesce et al., 2016). Stressors effects were assessed on leaf decomposition rates and a range of extracellular enzymatic activities associated (laccase, phenol oxidase, β-glucosidase, leucine-aminopeptidase and alkaline phosphatase), as well as on the biomass and structure of fungal and bacterial communities. Indirect effects of contamination were investigated on the feeding rates of the macroinvertebrate *Gammarus fossarum*, a shredder amphipod which represents the dominant macroinvertebrate species, in terms of biomass, in many lotic ecosystems (MacNeil et al., 1997). Macroinvertebrates were fed using leaves previously exposed for 40 days to each of the treatments described above.

We firstly hypothesized that the fungicide exposure (alone or in mixture with the herbicide) would significantly affect fungal communities associated with *Alnus* leaves, while the effects of the herbicide alone would be expected to be very limited due to the lack of direct toxicity to fungi and bacteria. In addition, we hypothesized that nutrient increase relative to the eutrophic condition could minimize the effects of pesticides on leaf-associated microbial communities and therefore mask pesticide effects on leaf palatability for the macroinvertebrate *Gammarus fossarum*.

## Materials and Methods

### Microbial Inoculum

Leaf-associated fungal and bacterial communities were obtained from the Couze d’Ardes River, a well preserved mesotrophic third-order forested stream, draining a basin surface area of 21476 ha (mostly forest and prairies) in the Puy-de-Dôme region (Centre France). Freshly fallen *Alnus glutinosa* (L.) Gaertn leaves were harvested in October 2016, brought back to the laboratory and dried at room temperature for 72 h. Leaves (ca. 3 g of dried leaves) were placed into three litter bags of 0.5 mm mesh size (l × w = 15 × 15 cm) and immersed in the upstream section of the Couze d’Ardes River during 4 weeks, from September 25 to October 26 2016, to allow microbial colonization. After in-stream colonization, litter bags were retrieved and transported back to the laboratory where leaves were cleaned with filtered stream water (0.2 μm), cut into small circles (1 cm in diameter) and placed into nine sterile 250 mL flasks (3 flasks per retrieved bag). Each flask was filled with 200 mL of stream water diluted to 1/10 with demineralized water and contained 15 pre-colonized leaf disks (Gessner and Chauvet, 1993). Fungal mycelia sporulation was achieved by incubations at 10°C under agitation at 180 rpm (Artigas et al., 2008). After 5 days, water suspension and leaf disks from the same replicates were pooled by transferring the content into three new sterile flasks (1 per replicate, 600 mL per flask) and used as inoculum for the subsequent microcosm experiment (75 mL of inoculum per stream channel).

### Experimental Design

The experiment was performed in microcosm under controlled conditions of temperature (20 ± 2°C) and photoperiod (13 h light: 11 h dark). In total, 24 artificial glass stream channels were set up (63 cm × 11 cm × 4 cm = l × w × h) following a 2 × 4 factorial design, with nutrient condition as first factor (mesotrophic and eutrophic) and pesticide treatment as second one (Control, TBZ, S-Met, and MIX) in triplicates. Each channel was independent and had a separate 20 L tank, filled with 10 L of water from the corresponding condition (see details below) which supplied water through an individual aquarium pump (MJ 750, NEWA, 1.5 L.min^-1^). Drilled ground water diluted to 1/3 with demineralized water was used for the experiment and distributed in two different 200 L tanks filled up to 100 L. Nutrient conditions were obtained by adding monobasic potassium phosphate (K_2_HPO_4_) and sodium nitrate (NaNO_3_) to reach nominal concentrations of 8 mg L^-1^ N-NO_3_ and 0.5 mg L^-1^ P-PO_4_ for the eutrophic condition and 0.8 mg L^-1^ N-NO_3_ and 0.05 mg L^-1^ P-PO_4_ for the mesotrophic one. Nutrient enriched water was then distributed into 8 smaller tanks of 30 L (4 for either mesotrophic and eutrophic conditions), and pesticide contaminated treatments were obtained by adding either TBZ, S-Met or both from a 5 mg L^-1^ stock solution diluted in water (Sigma–Aldrich, Germany) to reach nominal pesticide concentrations of 15 μg L^-1^ TBZ or/ and 15 μg L^-1^ S-Met in the corresponding condition. Those 30 L tanks were finally used to supply water to each triplicate channels per each pesticide treatment.

In each channel, a total of 15 bags containing *Alnus glutinosa* leaves was disposed as follows: 6 small bags containing each 60 leaf disks (1cm diameter) for biological analyses (extracellular enzymatic activities, fungal and bacterial biomass, fungal and bacterial community structure), 2 bags containing each 40 leaf disks (2 cm diameter) for *Gammarus* feeding rates, 1 bag containing 60 leaf disks (2 cm diameter) for adsorbed pesticides and leaf nutrient content analyses and 6 larger bags containing each 2 pre-weighted leaves for leaf mass loss determination. The experiment started in November 3 until December 13 2016 (6 weeks duration in total). After 4 days acclimation of leaves in all 24 channels under mesotrophic water (plus microbial inoculums) without pesticides, water from the channels was replaced to fit the eight experimental conditions described above. Samplings for biological analyses were performed every week by taking one of the small bags cited above and water was renewed weekly during the entire experiment using the above described procedure (6 water renewals during the whole experiment).

### Water and Leaves Characteristics

Physical and chemical parameters of stream water, including dissolved nutrients, TBZ, and S-Met concentrations were measured before and after each water renewal to evaluate nutrient and pesticide dissipation. Light was measured continuously for each condition using data loggers (HOBO^®^ Pendant Temperature/Light, Prosensor), whereas temperature, pH, conductivity and dissolved oxygen were measured in each channel before and after each water renewal using portable probes (WTW). Concentrations of orthophosphates (P-PO_4_), nitrates (N-NO_3_), nitrites (N-NO_2_) and ammonium (N-NH_4_) were determined using ionic chromatography (930 Compact IC Flex, Metrohm) following standard methods for anions (NF EN ISO 10304-1; AFNOR, 2009) and cations (NF EN ISO 14911; AFNOR, 1999). Dissolved organic carbon (DOC) concentrations were measured in water following standard method (NF EN 1484; AFNOR, 1997) using an elemental analyzer (Multi N/C 3100, Analytik Jena). Prior to analyze DOC, inorganic carbon was removal by purging acidified (pH = 1 with HCl) samples with a CO_2_-free gaz. Dissolved TBZ and S-Met concentrations were determined by direct injection of 20 μL of water samples previously filtered using a 0.2 μm polyester filter (MACHEREY-NAGEL) into an Ultra-High-Performance Liquid Chromatography (UHPLC) system (Shimadzu Nexera^®^) coupled to a triple quadrupole mass spectrometer (API 4000, LC/MS/MS system, AB Sciex). The chromatographic separation was performed on a Waters HSS T3 1.8 μm 2.1 × 100 mm column. Limit of quantification was 0.05 μg L^-1^ for both pesticides.

Chemical composition of *Alnus* leaves (C, N, and P content) and adsorbed pesticides (TBZ and S-Met) were assessed at the end of the microcosm experiment (Day 40). Total carbon and nitrogen of the leaves were measured by dry combustion using CNS elemental analyzer (Flash2000, ThermoFisher Scientific). Total phosphorus was analyzed after microwave-acid mineralization in *aqua regia* (NF EN ISO 15587-1; AFNOR, 2002) using inductively coupled plasma optical emission spectrometry (ICP-OES, 700 Series, Aglient Technologies) following standard method (EN ISO 11885, 2009). Results were obtained in mg per kg^-1^ of leaf dry mass (DM)^-1^ and converted into C:N, N:P, and C:P molar ratios. Adsorbed pesticides were extracted from leaves using the QuEChERS protocol (Payá et al., 2007) and pesticide were quantified using ultra-high performance liquid chromatography coupled to tandem mass spectrometry (UHPLC-MS-MS). Results were expressed in ng per g leaf DM^-1^ with a limit of quantification for both the TBZ and S-Met of 8 ng g leaf DM^-1^.

### Microbial Biomass and Community Structure

Ergosterol concentration was used as a proxy to estimate fungal biomass associated with leaves (Gessner and Schmitt, 1996). Briefly, lipids were extracted from previously lyophilized leaves (15 leaf disks of 1 cm in diameter per sample) after incubation in 0.14 M KOH methanol at 80°C. Extracts were purified, concentrated using solid-phase extraction (tC18 cartridges, Sep-Pak Vac RC, 500 mg, Waters) and then analyzed using a high pressure liquid chromatography system (Lachrom L-7400, Merck-Hitachi) equipped with an ODS-2 Hypersil column (250 × 4.6 mm, 5 μm particle diameter; Thermo Scientific). Ergosterol was detected at 282 nm according to its specific absorbance spectrum. Ergosterol concentration was converted into fungal carbon using the conversion factor proposed by Gessner and Chauvet (1993) and Baldy and Gessner (1997).

Bacterial biomass was estimated using bacterial density according to the protocol of Borrel et al. (2012). Briefly, one leaf disk (1 cm diameter) was sampled at each sampling time and for each channel and fixed into 1 mL of 2% paraformaldehyde (storage at 4°C) until analysis using a BD FACS Calibur flow cytometer (Becton Dickinson Bio-sciences) (see Pesce et al., 2016). Bacterial density was converted to bacterial biomass using the conversion factor of (Bratbak, 1985).

The structure of fungal and bacterial communities associated to *Alnus* leaves was assessed using Automated Ribosomal Intergenic Spacer Analysis (ARISA) as described in Artigas et al. (2012). Total DNA extraction was performed from 5 leaf disks of 1 cm using the FastDNA SPIN Kit for soil (MP Biomedicals, Santa Ana, CA, United States) according to the manufacturer’s instructions. Fungal community was characterized by amplification of the ITS1-5.8S-ITS2 region using the primers 2234C (5′-GTTTCCGTAGGTGAACCTGC-3′) and 3126T (5′-ATATGCTTAAGTTCAGCGGGT-3′). Bacterial community was characterized by amplification of the 16S-23S ribosomal intergenic spacer region using the primers S-D-Bact-1522-b-S-20 (5′-185 TGCGGCTGGATCCCCTCCTT-3′) and L-DBact-132-a-A-18 (5′-CCGGGTTTCCCCATTCGG-3′). PCR mixture and conditions are described in Baudoin et al. (2010). ARISA was performed using an Agilent 2100 Bioanalyzer with a DNA 1000 kit (Agilent Technologies) following the manufacturer’s instructions. ARISA profiles (**Supplementary Figures S3, S4**) were then analyzed using the Gelcompare2 software (Applied Maths, Belgium) in order to obtain a fungal and bacterial OTUs presence/absence matrix.

### Microbial Activity

Decay rates of *Alnus* leaves (*k*) were evaluated as the leaf mass loss during the experiment. At each sampling time and in each channel, 1 bag containing 2 pre-weighted *Alnus* leaves was sampled and brought to the oven (70°C for 48h) in order to obtain dry mass (DM). Decay rates were then obtained after fitting data to an exponential decay model according to the following equation: M_t_ = M_0_ e^-kt^, where M_0_ is the initial DM (g), M_t_ is the final DM (g) at time *t*, and *k* is the leaf decay coefficient (Petersen and Cummins, 1974).

Microbial enzymatic potential was estimated through the measurement of 5 extracellular enzyme activities: phenol oxidase (EC 1.14.18.1), laccase (EC 1.10.3.2), β-glucosidase (EC 3.2.1.21), alkaline phosphatase (EC 3.1.3.1) and leucine-aminopeptidase (EC). Phenol oxidase and laccase activities were measured from five leaf disks per sample, using 3,4-Dihydroxy-L-phenylalanine (L-DOPA, 1.5 mM final concentration) and 2,2′-Azino-bis(3-ethylbenzthiazoline-6-sulfonic acid (ABTS, 3 mM final concentration) respectively, in acetate buffer (pH 4.65, Sigma-Aldrich, St. Louis, MO, United States), as described in Sinsabaugh et al. (1994) and Johannes and Majcherczyk (2000). β-glucosidase, leucine-aminopeptidase and alkaline phosphatase activities were measured from 1 leaf disk per sample using the corresponding methylumbelliferyl and amido-4-methylcoumarin substrate analog (0.3 mM final concentration for each activity assay), as described in Artigas et al. (2012). After 1.5 h incubation in the dark and under agitation at 20°C, phenol oxidase and laccase activities were measured spectrophotometrically (460 nm and 415 nm, respectively) using an Ultrospec 2000 (Pharmacia Biotech, Trowbridge, United Kingdom) whereas β-glucosidase, leucine-aminopeptidase and alkaline phosphatase were determined fluorometrically (360/460 nm excitation/emission, Biotek synergy HT fluorometer) after addition of 0.3 mL glycine buffer (pH 10.4). All enzyme activity measurements were conducted under substrate saturating conditions. Area under the curve (AUC) was calculated for each enzyme and divided by the AUC for microbial biomass in order to obtain one value of integrated specific enzymatic activity for each nutrient condition and pesticide treatment. Activity values were expressed in μmol substrate h^-1^ mg of microbial C^-1^.

### *Gammarus fossarum* Feeding

Feeding rates of *Gammarus fossarum* were assessed as described in Pesce et al. (2016). Briefly, *Gammarus* were harvested from the River Le Pollon in France (45°57′25.8^′′^N 5°15′43.6^′′^E) and acclimatized to laboratory conditions as previously described (Coulaud et al., 2011). In the laboratory, organisms were kept in 30 L tanks continuously supplied with drilled groundwater (500 μS/cm) and under constant aeration for 10 days. Photoperiod (16 h light: 8 h dark) was maintained and the temperature was kept at 12 ± 1°C. Organisms were fed *ad libitum* with alder leaves (*Alnus glutinosa*), previously conditioned for 6 ± 1 days in water. At the end of the microcosm experiment, bags containing 2 cm leaf disks were recovered and displayed into plastic beakers (8 disks per beaker) filled with 500 mL of oxygenated water (12°C) and containing 12 *Gammarus* males with homogenous body size (∼10 mm). The experiment was performed in triplicate for each replicate (*n* = 9) and included a control (leaves without *G. fossarum*) to estimate leaf mass loss unrelated to *Gammarus* consumption. In parallel, daily water renewals were performed and dead *Gammarus* were recorded and removed if any to avoid bias in feeding rates estimation. Only 3 deaths occurred over the whole set of replicates (864 gammarids). The experiment was stopped after 28 h when leaf consumption achieved approximately 50% in at least one replicate, and the surface of remaining leaf disks was scanned numerically using SigmaScan^®^Pro v5.0 imaging software (Systat Software). Results were expressed in % of leaf consumed per *Gammarus* and per day and converted into ingested fungal C (mg Fungal C Gammarus ^-1^Day ^-1^) using the measurements of fungal C on leaves (see above).

### Statistical Analyses

Differences between nutrient conditions and between pesticide treatments for water physical and chemical characteristics (pH, oxygen, conductivity, temperature, TBZ and S-Met), leaf composition (C:N, C:P, and N:P, adsorbed TBZ and S-Met), decomposition rates, specific enzymatic activities and leaf palatability for macroinvertebrates were assessed using a Kruskal–Wallis non parametric test followed by pairwise comparison test using Tukey contrast (*P* < 0.05). In addition, fungal and bacterial biomass colonization on *Alnus* leaves was fitted to a logistic growth model using the following equation:

$$y=\frac{A}{1+\frac{A-y0}{y0}e^{-4W\;\max x/A}}$$

Where *y*0 is the fungal biomass (mg of fungal carbon g Leaf DM^-1^) at day 0, *A* is the fungal biomass at day 40 and *Wmax* is the maximum growth rate (day^-1^). Parameters from the model were then compared within each-other using the Kruskal–Wallis non-parametric test followed by pairwise comparison test using Tukey contrast (*P* < 0.05). In addition, two non-metric dimensional scaling (NMDS) for either bacteria or fungi were used to assess communities’ structure based on Bray-Curtis dissimilarity matrices. First NMDS was performed including all sampling times, whereas second NMDS was performed on last sampling time only (day 40). Each NMDS was accompanied by permutational multivariate analysis of variance (PERMANOVA) using nutrient condition as first factor and treatment as second one. All statistical analyses were computed using the R software and NMDS and PERMANOVA analyses were performed using metaMDS and Adonis functions from the vegan package (Oksanen et al., 2008) followed by pair-wise *post hoc* multiple comparisons.

## Results

### Water and Leaves Characteristics

Average initial phosphate, nitrate and S-Met concentrations measured in water right after each water renewal were relatively close to the expected nominal concentrations (**Table 1**). In contrast, initial TBZ concentrations were about one-third lower than expected. No statistical differences were observed for both initial TBZ and S-Met concentrations between treatments (alone or MIX) and the subsequent water renewals. Similarly, no statistical differences were observed for initial N-NO_3_ and P-PO_4_ concentrations within each nutrient condition and between further water renewals. In addition, water temperature, dissolved oxygen concentration and pH displayed similar values between treatments (Kruskal–Wallis, *P* > 0.05, **Table 1**), while water conductivity displayed higher values in eutrophic conditions (Kruskal–Wallis, *P* < 0.001) probably because of the higher nutrient (phosphate and nitrate) concentrations.

**Table 1:** Water physical and chemical characteristics measured after each water renewals in experimental stream channels exposed to fungicide (TBZ), herbicide (S-Met), both fungicide + herbicide (MIX) or non-exposed (Ctrl) in either mesotrophic or eutrophic nutrient condition.

	Mesotrophic				Eutrophic			
	Ctrl	TBZ	S-Met	MIX	Ctrl	TBZ	S-Met	MIX
P.PO4 (mg L^-1^)	0.05 ± 0.01				0.46 ± 0.01			
N.NO3 (mg L^-1^)	0.66 ± 0.01				7.89 ± 0.03			
TBZ water (μg L^-1^)	<0.05	9.87 ± 0.30	<0.05	11.39 ± 0.85	<0.05	10.67 ± 0.75	<0.05	11.64 ± 0.86
S-Met water (μg L^-1^)	<0.05	<0.05	16.26 ± 1.22	14.28 ± 0.54	<0.05	<0.05	17.21 ± 1.39	15.79 ± 0.96
TBZ leaf (μg gDM^-1^)	<0.008	1.22 ± 0.13	<0.008	1.16 ± 0.09	<0.008	0.68 ± 0.04	<0.008	0.71 ± 0.06
S-Met leaf (μg gDM^-1^)	<0.008	< 0.008	1.37 ± 0.19	1.05 ± 0.10	<0.008	<0.008	0.64 ± 0.05	0.50 ± 0.04
Temperature (°C)	19.9 ± 0.3	20.1 ± 0.3	18.4 ± 1.8	20.2 ± 0.2	20.2 ± 0.2	20.3 ± 0.2	20.4 ± 0.2	20.3 ± 0.2
Conductivity (μS)	167 ± 1	170 ± 2	168 ± 1	168 ± 1	208 ± 9^∗^	205 ± 9^∗^	206 ± 9^∗^	205 ± 9^∗^
Oxygen (mg L^-1^)	8.3 ± 0.1	8.2 ± 0.1	8.1 ± 0.1	8.2 ± 0.1	8.3 ± 0.2	8.3 ± 0.2	8.6 ± 0.4	8.4 ± 0.2
pH	7.7 ± 0.1	7.7 ± 0.1	7.7 ± 0.1	7.8 ± 0.1	7.8 ± 0.1	7.9 ± 0.1	7.9 ± 0.2	7.9 ± 0.2

*Values are means ± standard error of the mean over the all experiment (*n* = 5 for pesticides and *n* = 6 for nutrients, temperature, conductivity, oxygen, and pH). Asterisks indicates significant differences between nutrient conditions for the considered treatment (Kurskal–Wallis, *P* < 0.05)*.

**FIGURE 1:**
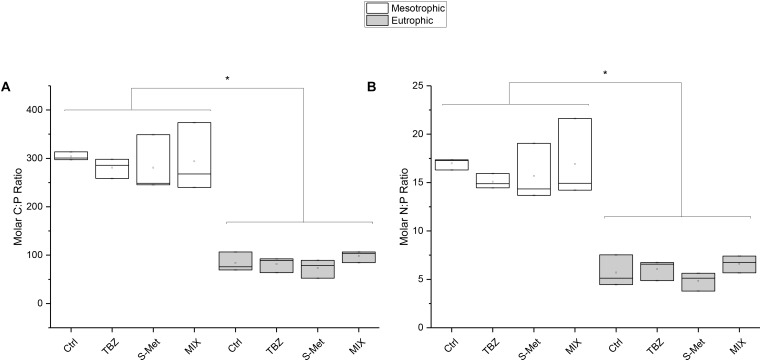
Molar C:P **(A)** and N:P **(B)** ratios recorded at the end of the experiment in leaves exposed to fungicide (TBZ), herbicide (S-Met), both fungicide + herbicide (MIX) or non-exposed (Ctrl), in either mesotrophic (white fill) or eutrophic (gray fill) nutrient condition. Values are median + quartiles 1 and 3 (*n* = 3). Differences between nutrient conditions are represented by an asterisk (Kruskal–Wallis, *P* < 0.05).

Nutrient dissipation, calculated as the difference after and before each water renewal in stream channels (once per week), revealed that both nitrate and phosphate were completely dissipated in the mesotrophic condition (**Table 2**), but not in the eutrophic one (average dissipation percentage of 65.4% for N-NO_3_ and 93.1% for P-PO_4_). In the eutrophic condition, P-PO_4_ dissipation was greater than that of N-NO3 revealing that leaf-associated microbial community’s used more P than N from water. This difference was also reflected in leaves nutrient content at the end of the experiment (Kruskal–Wallis, *P* < 0.01, **Figure 1**). Specifically, lower C:P and N:P ratios were recorded in eutrophic channels, which show the greater P accumulation in leaves and therefore confirms the greater P dissipation from water. Pesticide treatments did not significantly influence N-NO_3_ and P-PO_4_ dissipation (Kruskal–Wallis, *P* = 0.18 and *P* = 0.13 respectively), although nitrate dissipation for the eutrophic condition was about 13–22% greater in the MIX in comparison with Ctrl, and S-Met and TBZ alone (**Table 2**).

**Table 2:** Average percentage of nutrients and pesticides dissipation as well as adsorbed pesticides on leaves recorded at the end of the experiment in experimental stream channels exposed to fungicide (TBZ), herbicide (S-Met), both fungicide + herbicide (MIX) or non-exposed (Ctrl) in either mesotrophic or eutrophic nutrient condition.

	Mesotrophic				Eutrophic			
	Ctrl	TBZ	S-Met	MIX	Ctrl	TBZ	S-Met	MIX
P-PO4	100	100	100	100	93.80 ± 2.05	98.01 ± 1.13	88.26 ± 2.23	92.46 ± 3.24
N-NO3	100	100	100	100	64.99 ± 5.90^∗^	57.98 ± 4.84^∗^	63.89 ± 7.37^∗^	74.53 ± 0.87^∗^
TBZ	–	38.87 ± 2.88	–	45.80 ± 2.12	–	43.56 ± 2.93	–	51.19 ± 1.28
S-Met	–	–	54.68 ± 0.99	50.16 ± 1.89	–	–	56.45 ± 0.77	69.40 ± 0.39
Adsorbed TBZ (μg g of leaf DM^-1^)	–	1.22 ± 0.13	–	1.17 ± 0.09	–	0.68 ± 0.04^∗^	–	0.72 ± 0.06^∗^
Adsorbed S-Met (μg g of leaf DM^-1^)	–	–	1.37 ± 0.19	1.05 ± 0.10	–	–	0.64 ± 0.05^∗^	0.50 ± 0.04^∗^

*Values are means ± standard error of the mean over the all experiment including all replicates (*n* = 15 for pesticides and nutrients dissipation in water and *n* = 3 for adsorbed TBZ and S-Met on leaves). Asterisks indicates significant differences between nutrient conditions for the considered treatment (Kurskal–Wallis, *P* < 0.05)*.

Concerning pesticides in water, about 39–70% of each pesticide were dissipated every week in all pesticide-treated channels (**Table 2**). For each pesticide, no differences in dissipation were observed between nutrient conditions and pesticide treatments (i.e., Alone vs. MIX) with some exceptions. In eutrophic channels, S-Met dissipation was slightly greater in the MIX treatment (69% dissipation) compared to S-Met alone (56%) (Kruskal–Wallis, *P* < 0.05). Pesticide sorption was assessed at the end of the experiment and only a small part of TBZ and S-Met weekly supplied to channels was adsorbed onto *Alnus* leaves (less than 5%, **Table 2**). Comparatively, pesticide adsorption was higher in mesotrophic channels compared with the eutrophic ones (Kruskal–Wallis, *P* < 0.05). Exposure of pesticides alone or in MIX did not have an effect on their adsorption to leaves.

### Microbial Leaf-Litter Decomposition

*Alnus* leaves decomposition displayed differences between both nutrient conditions (Kruskal–Wallis, *P* < 0.001) and pesticide treatments (Kruskal-Wallis, *P* < 0.01, **Table 3** and **Supplementary Table S1**). On average, leaf decay rates in eutrophic channels were 34% higher compared to those in mesotrophic channels. In addition, decay rates were on average 17% higher in control than in pesticide-treated channels (including TBZ, S-Met, and MIX) in the eutrophic condition, whereas no differences between pesticide treatments and control were observed under mesotrophic conditions. No differences were observed for *Alnus* decay rates in TBZ and S-Met alone compared to MIX, regardless of the nutrient condition.

**Table 3:** Alnus leaves decomposition rates (K, means ± standard error of the mean) expressed in day^-1^ measured in experimental stream channels exposed to fungicide (TBZ), herbicide (S-Met), both fungicide + herbicide (MIX) or non-exposed (Ctrl), in either mesotrophic or eutrophic nutrient condition.

	Ctrl		+TBZ		+S-Met		+MIX	

	*K*	*R*^2^	*K*	*R*^2^	*K*	*R*^2^	*K*	*R*^2^
Eutrophic	13.34 × 10^-3^ ± 1.52 × 10^-3^	0.94	**11.19 × 10^-3^** ± 1.23 × 10^-3^	0.95	**11.39 × 10^-3^** ± 1.34 × 10^-3^	0.94	**10.48 × 10^-3^** ± 1.27 × 10^-3^	0.93^∗^
Mesotrophic	8.81 × 10^-3^ ± 1.91 × 10^-3^	0.81	8.69 × 10^-3^ ± 1.79 × 10^-3^	0.83	7.49 × 10^-3^ ± 1.52 × 10^-3^	0.83	7.38 × 10^-3^ ± 1.39 × 10^-3^	0.85

*Values were obtained by fitting average leaf mass loss (in %) over the experiment for each treatment to an exponential decay model and goodness of fit was represented by *R*^2^. Bold values indicate significant differences between treatments and control within nutrient condition whereas asterisk indicates significant differences between nutrient conditions*.

**FIGURE 2:**
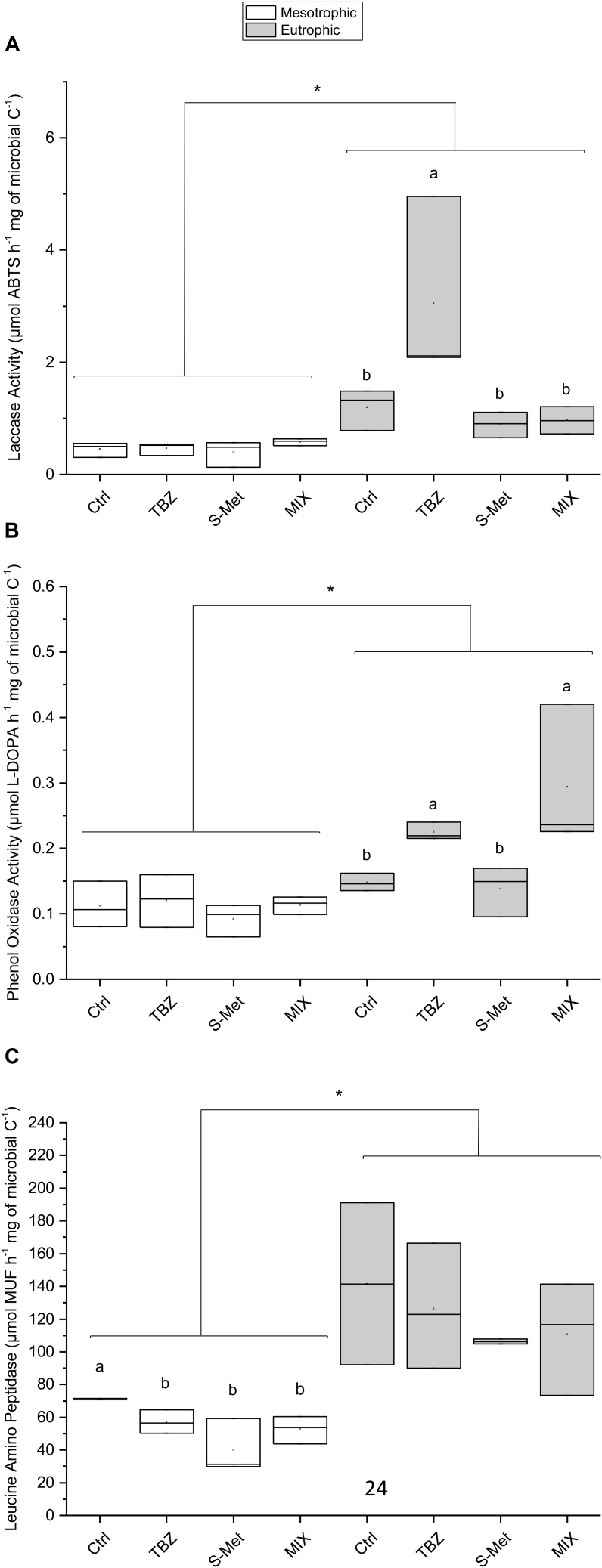
Integrated extracellular specific laccase **(A)**, phenol oxidase **(B)**, and leucine aminopeptidase activities **(C)** measured in experimental stream channels exposed to fungicide (TBZ), herbicide (S-Met), both fungicide + herbicide (MIX) or non-exposed (Ctrl), in either mesotrophic (white fill) or eutrophic (gray fill) nutrient condition. Values are median + quartiles 1 and 3 (*n* = 3). Differences between nutrient conditions are represented by an asterisk (Kruskal–Wallis, *P* < 0.05) whereas differences between treatments are represented by subscript (a > b).

Specific extracellular enzymatic activities (i.e., cumulated enzymatic activity corrected by cumulated microbial biomass) displayed variation between the tested conditions and the enzyme considered (**Figure 2**). Specifically, differences in ligninolytic (laccase and phenol oxidase, **Figures 2A,B**) and peptidase (leucine-aminopeptidase, **Figure 2C**) activities were observed between both nutrient conditions and pesticide treatments, whereas no between- treatment difference were observed for β-glucosidase and alkaline phosphatase (data not shown). Ligninolytic activities (phenol oxidase and laccase) were higher in eutrophic than in mesotrophic conditions (Kruskal–Wallis, *P* < 0.001 each). Exclusively for the eutrophic condition, phenol oxidase activity was enhanced in both TBZ and MIX treatments (pairwise, *P* < 0.001 each) while laccase activity increased in the presence of TBZ (pairwise, *P* < 0.001), but not in the MIX treatment (pairwise, *P* = 0.90). As for ligninolytic activities, leucine aminopeptidase was higher in the eutrophic condition (Kruskal–Wallis, *P* < 0.001) but effects of pesticide treatments were only observed in the mesotrophic condition. Under the mesotrophic nutrient condition, lower peptidase activity was recorded in all pesticide exposed channels (including TBZ, S-Met, and MIX) compared to control (pairwise, *P* < 0.05 each) with a reduction that was more important in channels exposed to S-met (average reduction of 44.8% compared to control).

### Microbial Biomass and Communities’ Structure

Fungal biomass colonizing *Alnus* leaves displayed differences between nutrient conditions (Kruskal–Wallis, *P* < 0.001). Globally, higher fungal biomass was accumulated at the end of the experiment in the eutrophic condition compared to the mesotrophic one (A, **Table 4** and **Supplementary Figure S1** and **Table S2**), despite overall lower fungal growth rate (*Wmax*) under eutrophic conditions than in mesotrophic conditions. Stationary phase for eutrophic channels (**Supplementary Figure S1**) was reached around 35 days or more than 40 days for the control treatment, except for the MIX treatment which occurred much earlier (around 25 days). No differences between pesticide treatments were observed in terms of maximal biomass (*A*, **Table 4**). However, maximal growth rate in control channels displayed lower values in comparison with the pesticides exposed ones (*Wmax*, pairwise, *P* = 0.001). In contrast with eutrophic channels, stationary phase for mesotrophic channels (**Supplementary Figure S1**) was reached much earlier (around 10 days). Again, no differences were observed under those nutrient conditions between pesticide treatments for maximal biomass neither for maximal growth rates.

**Table 4:** Parameters extracted from the measured fungal biomass after fitting to a logistic growth model in experimental stream channels exposed to fungicide (TBZ), herbicide (S-Met), both fungicide + herbicide (MIX) or non-exposed (Ctrl), in either mesotrophic or eutrophic nutrient condition.

		Y_0_ (mg of fungal C gDM^-1^)	A (mg of fungal C gDM^-1^)	W_max_ (Day^-1^)	*R*^2^	*p*-value
Mesotrophic	Ctrl	7.28 ± 0.60	18.86 ± 0.27	2.82 ± 0.38	0.99	1.08^E^-6
	TBZ	7.35 ± 1.62	20.05 ± 0.74	2.49 ± 0.79	0.93	4.60^E^-5
	S-Met	7.28 ± 2.25	18.65 ± 1.01	5.75 ± 13.85	0.84	1.97^E^-4
	MIX	7.62 ± 1.60	20.54 ± 0.77	1.95 ± 0.64	0.93	4.35^E^-5
Eutrophic	Ctrl	10.26 ± 1.71	**35.61 ± 3.26**	**0.89 ± 0.18**	0.96	5.40E-5
	TBZ	9.36 ± 2.44	**33.16 ± 2.26**	**1.35 ± 0.39^∗^**	0.93	1.45E-4
	S-Met	9.34 ± 3.40	**34.58 ± 3.37**	**1.35 ± 0.52^∗^**	0.89	5.18E-4
	MIX	8.88 ± 2.22	**30.57 ± 1.91**	**1.32 ± 0.38^∗^**	0.94	1.20E-4

*Values are means ± standard error of the mean returned by the origin software (*n* = 3). Y_*0*_ represent the initial fungal biomass (day 0), A represent the maximal fungal biomass (day 40) and Wmax represent maximal fungal growth rate. Values in bold represent differences between nutrient conditions (Kruskal–Wallis, *P* < 0.05) and asterisk represent differences between treatments and control within each nutrient conditions (Tukey, *P* < 0.05)*.

**FIGURE 3:**
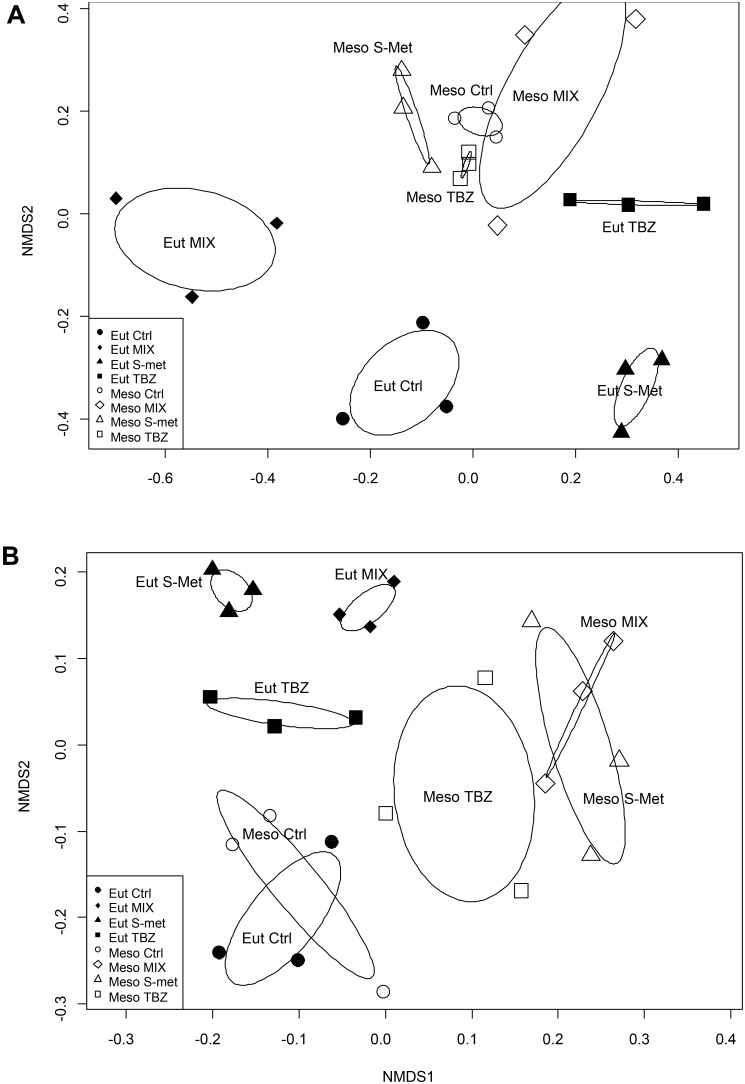
Non metric dimensional scaling (NMDS) performed on samples at the end the experiment (day 40) representing fungal **(A)** and bacterial **(B)** community structure with ellipses representing standard deviation of samples within each treatment and nutrient condition.

The fungal community structure appeared to mostly vary between sampling dates rather than according to nutrient and/or pesticide treatments (**Supplementary Figure S3A**). However, nutrient conditions also influenced the structure of these communities leading to significant differences between mesotrophic and eutrophic conditions the end of the study (PERMANOVA, *P* < 0.001, **Figure 3A**). Here, no differences were observed in terms of OTU’s richness but in terms of OTUs replacement leading to changes in community structure between nutrient conditions. Differences were also observed at the end of the experiment between pesticide treatments regardless of the nutrient condition (PERMANOVA, *P* < 0.001) although such differences were much more marked for the eutrophic condition (**Figure 3A**). Again, differences were explained by OTUs replacement rather than by shifts in OTUs richness.

Bacterial biomass was very low in comparison with that of fungi, representing less than 1% of the total microbial biomass colonizing *Alnus* leaves (**Supplementary Figure S2**). Similarly to fungi, bacteria displayed higher biomass values in the eutrophic condition compared to the mesotrophic one, although differences were less marked than for fungi (Kruskal–Wallis, *P* < 0.001). Again, no differences were observed between pesticide treatments regardless of the nutrient condition. As for fungi, the structure of bacterial communities appeared to be mostly influenced by the sampling time (**Supplementary Figure S3B**). Significant differences were also observed between nutrient conditions at the end of the study (PERMANOVA, *P* < 0.001, **Figure 3B**), although these differences were less marked than those for fungi. In addition, differences were observed between pesticide treatments regardless of the nutrient condition (PERMANOVA, *P* < 0.001) although still less marked in the case of the mesotrophic condition than the eutrophic one (**Figure 3B**). As for fungi, differences between nutrient conditions and pesticide treatments were mostly explained by OTUs replacement. However, control for both mesotrophic and eutrophic condition displayer lower OTUs richness compared to pesticides exposed treatments (both TBZ, S-Met and MIX).

### Macroinvertebrate Feeding Activity

**FIGURE 4:**
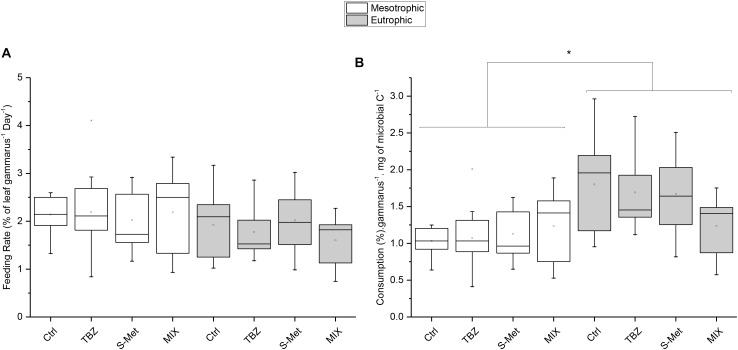
*Gammarus fossarum* feeding rates expressed in % of leaf ingested per Gammarus and **(A)** and mg of fungal carbon ingested per Gammarus **(B)** for leaves exposed to fungicide (TBZ), herbicide (S-Met), both fungicide + herbicide (MIX) or non-exposed (Ctrl), in either mesotrophic (white fill) or eutrophic (gray fill) nutrient condition. Values are median + quartiles 1 and 3 (*n* = 9). Differences between nutrient conditions are represented by an asterisk (Kruskal–Wallis, *P* < 0.05).

*Gammarus* feeding rates expressed as percentage of leaf consumption per individuals were neither affected by nutrients nor by pesticide treatments (Kruskal–Wallis, *P* = 0.13 and *P* = 0.73; respectively) (**Figure 4A**). Nevertheless, feeding rates corrected by mg of fungal carbon ingested per individual (**Figure 4B**) were significantly higher in eutrophic conditions compared to the mesotrophic ones (Kruskal–Wallis, *P* < 0.01). No statistical differences were observed between pesticide treatments within each nutrient condition, although values for corrected feeding rates seemed substantially lower in the MIX treatment (TBZ + S-Met) compared to other pesticide treatments (and especially control) under eutrophic conditions (**Figure 4B**).

## Discussion

In the present study, we assessed the effects of the interaction between nutrients (Nitrogen and Phosphorus) and pesticides (TBZ and S-Met, alone or in mixture) on the structure and activity of heterotrophic microbial communities associated with leaf litter. As already observed by Gulis and Suberkropp (2003), nutrients appeared as the main factor driving the activity and shaping the biomass and structure of the studied communities. However, significant effect of the pesticide treatments were observed, although rather minor in comparison with those of nutrients.

In mesotrophic condition, almost no effect of the pesticide treatments were observed on microbial communities, which contrast with our first hypothesis and with previous studies assessing the ecotoxicological effects of similar TBZ concentrations (Bundschuh et al., 2011; Artigas et al., 2012; Donnadieu et al., 2016). Only leucine aminopeptidase activity was slightly lower in treated channels than in control ones suggesting a potential direct but non-specific effects of pesticides’ toxicity on bacteria, known as the main producer of this enzyme (Romaní et al., 2006). This lack of microbial functional and structural response to TBZ might be explained by the fixed experimental conditions. Indeed, every week all dissolved nutrients were rapidly consumed by microorganisms. Hence, microbial communities may have suffered chronic nutrient deficiency in the mesotrophic channels, limiting biomass accumulation and possibly masking pesticide effects in our study.

Significant effects of the pesticide treatments were observed in eutrophic conditions, which contrast with our second hypothesis as we expected nutrient availability to buffer the effects of pesticide toxicity in leaf-associated microbial communities (Aristi et al., 2016; Fernández et al., 2016). The slight decrease in *Alnus* decay rates exposed to TBZ (-16%), S-Met (-15%) and MIX (-22%) might suggests that nutrient concentrations (i.e., N-NO_3_ and P-PO_4_) were not sufficient to compensate pesticide toxicity. Another explanation could be that co-occurrence between pesticides and high nutrients concentrations may have negative effects on microbial decomposition. In the study of Fernandes et al. (2009) assessing the interaction between a range of zinc (0.03 mg L^-1^, 0.98 mg L^-1^, and 9.8 mg L^-1^) and phosphate (0.05 mg L^-1^, 0.2 mg L^-1^, and 0.5 mg L^-1^) concentrations, strongest inhibition in *Alnus* decomposition were observed in leaves exposed to both zinc and high phosphate concentrations. Although mechanisms behind remain unclear, the author explained this by changes in fungal community structure. In our study, the changes in both fungal and bacterial community structure exposed to pesticides might explain the observed differences in terms of decomposition activity (Swan and Palmer, 2004; Gessner et al., 2010). Similarly, such changes in fungal community structure might explain the lower fungal growth rates in the control channels than in the pesticide treated ones (Zubrod et al., 2015). Since specific traits of certain species are known to have a greater influence on leaf decomposition (Duarte et al., 2006), high nutrient exposure in our study may have increased the contribution of some fungal species, which were at the same time more sensitive to pesticide exposure. However, this is a hypothesis that needs to be tested. Overall, our results show that nutrients exacerbated the effects of pesticides on leaf decay rates, and that the previously described compensatory effect of nutrients on pesticide toxicity (Fernández et al., 2016; Gardeström et al., 2016) is probably toxicant and/or concentration dependent and does not always apply on aquatic microbial communities.

Interestingly, TBZ and S-met in the MIX treatment displayed different kind of interactions in eutrophic channels in our study. Specifically, antagonistic-type interaction was observed between those two compounds on leaf decay rates as no differences were observed between TBZ, S-Met and MIX treatments. Similarly, the fact that laccase activity was stimulated in channels exposed to TBZ but not in channels exposed to the MIX (TBZ + S-Met) in the eutrophic condition also highlight interaction between those two compounds on laccase activity. The observed effects on laccase activity suggest that perhaps (i) MIX exposure had negative effect on specific microorganisms producers of laccase or (ii) that antagonistic-type interaction occurred between TBZ and S-Met on this enzymatic activity. On the contrary, no interaction between TBZ and S-Met was observed on phenol oxidase activity. Regarding the literature, ligninolytic enzyme activity stimulation has been linked to toxicity mitigation of phenolic molecules (Baldrian, 2006; Sinsabaugh, 2010; Karas et al., 2011). Thus, the observed stimulation in TBZ treatments (for both phenol oxidase and laccase) could reflect detoxification mechanisms set up by fungi, possibly resulting in TBZ transformation in order to reduce its toxicity (Junghanns et al., 2005; Artigas et al., 2017). Whatever the hypothesis considered, our results show that the interaction between different pesticide compounds, even if they present low potential threat to non-target organisms, may impair the ability of microbial communities to display proper stress response (Dawoud et al., 2017).

The interaction between nutrient conditions and pesticide treatments (TBZ and S-Met alone) did not affect shredder feeding rates. In the context of TBZ exposure, this result contrasts with the study of Bundschuh et al. (2011) but is consistent with that of Pesce et al. (2016). These inter-study differences may be explained by differences in TBZ concentrations supplied, which were substantially higher in the study of Bundschuh et al. (2011) (50 and 500 μg L^-1^) than in the study of Pesce et al. (2016) and the present one. The lack of effect of previous exposure of leaves to pesticides on feeding rates, and that whatever the tested treatment, can be explained by the lack of significant differences in term of fungal biomass between pesticide treatments, which is one of the main parameter influencing *Gammarus* feeding (Bundschuh et al., 2011; Zubrod et al., 2011). Besides, only small quantities of pesticides were found adsorbed on the exposed leaves, thus probably resulting in a low toxic effect to *Gammarus* macroinvertebrates. Overall, our results show that indirect effects of environmentally relevant concentrations of pesticides on leaf palatability for macroinvertebrates can be negligible.

## Conclusion

The present experiment showed that nutrients were the main parameter driving the structure and functioning of heterotrophic microbial communities colonizing *Alnus* leaf litter. However, we found that environmentally relevant concentrations of a fungicide and a herbicide, alone and/or in mixture, were sufficient to induce changes in the structure of microbial communities leading to a slight decrease in their decomposition activity, although no effects on leaf palatability for macroinvertebrates were observed. Interestingly, such effects were only visible under eutrophic conditions, suggesting antagonistic-type interaction between nutrients and xenobiotic on leaf decomposition.

## Author Contributions

ClM, JA, and SP designed the experimental design for this study. FR, ClM, JA, and SP participated in the redaction and discussion of the article. ChM performed all pesticides measurements (in water and adsorbed with leaves) whereas MM performed all nutrients measurements (carbon, nitrogen, and phosphorus in water and adsorbed with leaves). AC took care of the palatability tests for the macroinvertebrates. All authors were involved in the critical reading of the paper, with special contribution of ClM, JA, and SP.

## Conflict of Interest Statement

The authors declare that the research was conducted in the absence of any commercial or financial relationships that could be construed as a potential conflict of interest. The reviewer BA-B and handling Editor declared their shared affiliation.
