# Supplementary material for: Interactive Effects of Pesticides and Nutrients on Microbial Communities Responsible of Litter Decomposition in Streams
Source: Front Microbiol. 2018 Oct 17;9:2437. doi: 10.3389/fmicb.2018.02437 (PMC6199466; doi:10.3389/fmicb.2018.02437)
Supplement: Supplementary file 1 [file Data_Sheet_1.docx]

**Supplementary Material**

Figure 1: Fungal biomass expressed in mg of carbon per g of leaf DM in experimental stream channels exposed to fungicide (TBZ), herbicide (S-Met), both fungicide + herbicide (MIX) or non-exposed (Ctrl), in either mesotrophic (grey) or eutrophic (black) nutrient condition. Lines represents fitting to a logistic growth model whose parameter can be found in table 4. Values are mean + standard error of the mean (n = 3) and lines represents fitting to a logistic growth model (see material and methods). Asterisk represent differences between nutrient conditions (Tukey, *P* < 0.05).

Figure 2: Bacterial biomass expressed in mg of carbon per g of leaf DM in experimental stream channels exposed to fungicide (TBZ), herbicide (S-Met), both fungicide + herbicide (MIX) or non-exposed (Ctrl), in either mesotrophic (grey) or eutrophic (black) nutrient condition. Values are mean + standard error of the mean (n = 3). Asterisk represent differences between nutrient conditions (Tukey, *P* < 0.05).

Figure 3: Non metric dimensional scaling (NMDS) performed on all samples including all sampling times and representing fungal (A) and bacterial (B) community structure with ellipses representing standard deviation of samples for the time. Samples from eutrophic channels are represented in bold, whereas samples from mesotrophic ones are represented in italic.

A

B

Figure 4: ARISA (Automated Ribosomal Intergenic Spacer Analysis) profiles obtained for fungal (A) and bacterial (B) communities at T40.

A


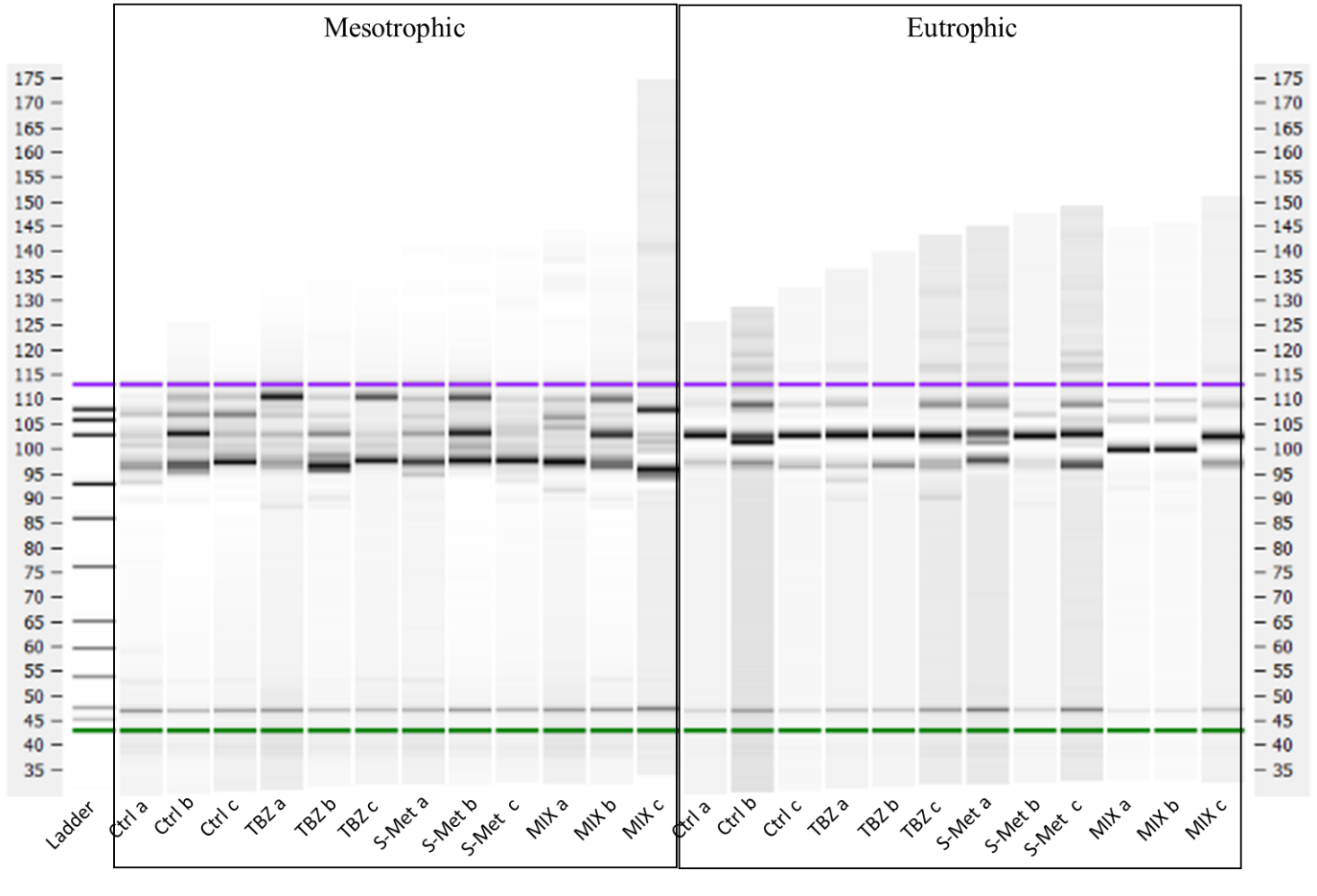


B


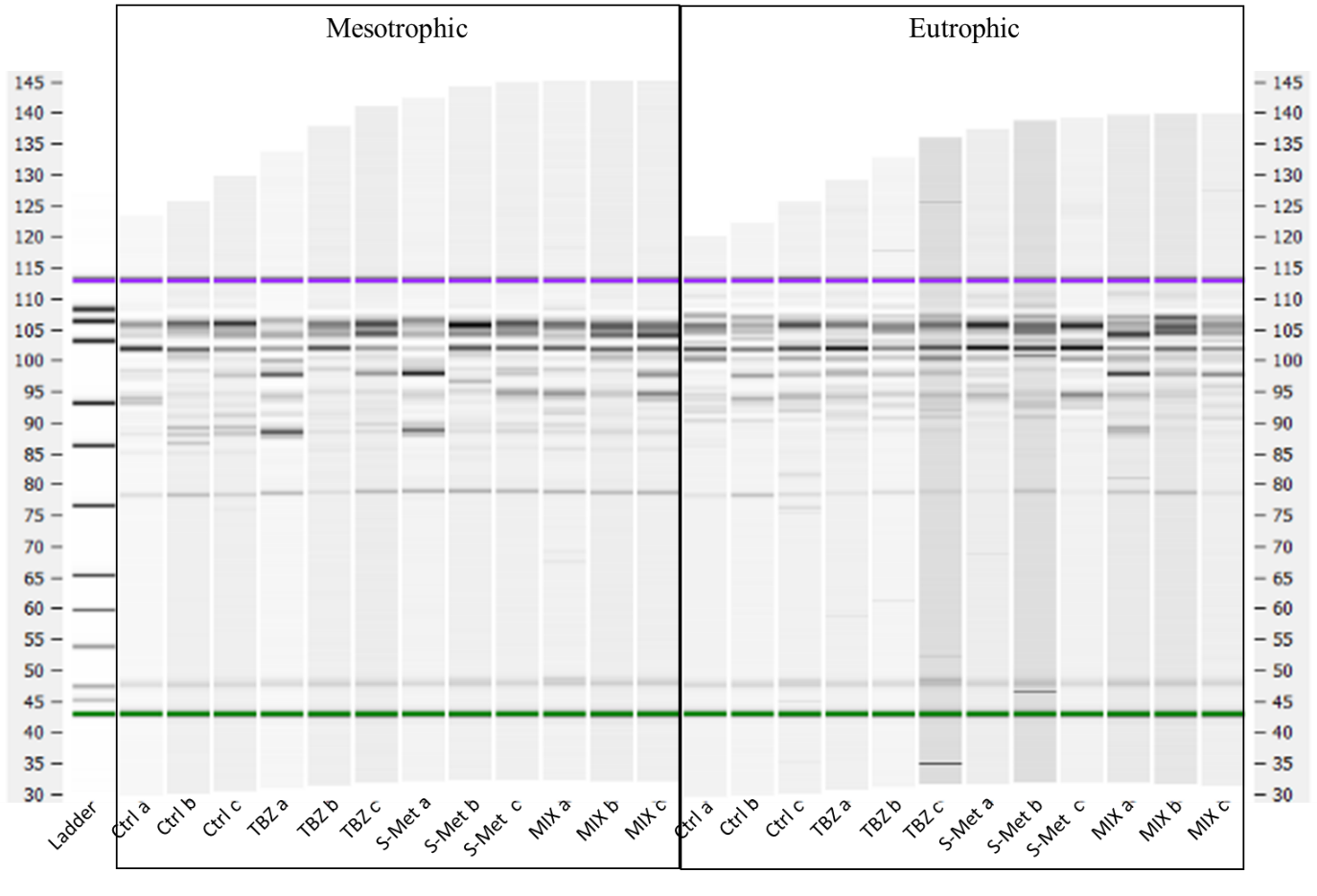


Table 1: Average remaining dry mass of Alnus leaves (in %) used for the fitting procedure and measured in experimental stream channels exposed to fungicide (TBZ), herbicide (S-Met), both fungicide + herbicide (MIX) or non-exposed (Ctrl), in either mesotrophic or eutrophic nutrient condition.

|  | Mesotrophic | Eutrophic |  |  |  |  |  |  |
| --- | --- | --- | --- | --- | --- | --- | --- | --- |
| Time (days) | Ctrl | TBZ | S-Met | MIX | Ctrl | TBZ | S-Met | MIX |
| 0 | 100 | 100 | 100 | 100 | 100 | 100 | 100 | 100 |
| 4 | 84.00905 | 83.76125 | 85.58392 | 86.48583 | 87.36658 | 85.9886 | 87.17876 | 90.27008 |
| 11 | 78.97287 | 78.93959 | 83.40579 | 82.04049 | 84.66865 | 84.31554 | 82.53983 | 83.81644 |
| 18 | 75.55303 | 80.46878 | 78.44845 | 81.58783 | 80.3215 | 77.85278 | 77.74488 | 80.33156 |
| 25 | 76.16988 | 74.35288 | 77.15282 | 77.68286 | 71.80628 | 70.02546 | 67.56479 | 75.76603 |
| 32 | 73.54492 | 69.12353 | 76.57678 | 74.70157 | 66.59252 | 67.92357 | 68.66945 | 74.26985 |
| 40 | 64.02093 | 67.80269 | 69.42975 | 70.97451 | 52.35511 | 61.94293 | 62.05282 | 60.02563 |

Table 2: Average fungal biomass colonizing Alnus leaves (in mg of fungal C per gDM) used for the fitting procedure and measured in experimental stream channels exposed to fungicide (TBZ), herbicide (S-Met), both fungicide + herbicide (MIX) or non-exposed (Ctrl), in either mesotrophic or eutrophic nutrient condition.

|  | Mesotrophic | | | | Eutrophic | | | |
| --- | --- | --- | --- | --- | --- | --- | --- | --- |
| Time (days) | Ctrl | TBZ | S-Met | MIX | Ctrl | TBZ | S-Met | MIX |
| 0 | 7.28184 | 7.28184 | 7.28184 | 7.28184 | 7.28184 | 7.28184 | 7.28184 | 7.28184 |
| 4 | 16.44622 | 16.38867 | 18.44028 | 15.58348 | 15.93228 | 16.7107 | 17.72412 | 15.97419 |
| 11 | 18.97615 | 18.59193 | 14.85667 | 18.46485 | 20.47482 | 22.69966 | 21.29056 | 21.59514 |
| 18 | 19.71642 | 20.15524 | 19.1345 | 18.51965 | 25.43287 | 28.09981 | 27.07358 | 25.487 |
| 25 | 18.82591 | 20.42543 | 18.95344 | 21.95523 | 27.47612 | 33.96279 | 39.15522 | 33.64912 |
| 32 | 18.70557 | 22.45179 | 19.40803 | 21.64307 | 32.46375 | 28.73702 | 33.89941 | 29.80131 |
| 40 | 18.03808 | 18.35064 | 20.87283 | 21.19883 | 35.04788 | 35.6956 | 31.10662 | 28.94499 |
